# Probiotics Partly Suppress the Impact of Sugar Stress on the Oral Microbiota—A Randomized, Double-Blinded, Placebo-Controlled Trial

**DOI:** 10.3390/nu15224810

**Published:** 2023-11-17

**Authors:** Christine Lundtorp Olsen, Laura Massarenti, Vincent Frederik Dahl Vendius, Ulvi Kahraman Gürsoy, Annina Van Splunter, Floris J. Bikker, Mervi Gürsoy, Christian Damgaard, Merete Markvart, Daniel Belstrøm

**Affiliations:** 1 Section for Clinical Oral Microbiology, Department of Odontology, Faculty of Health and Medical Sciences, University of Copenhagen, 2200 Copenhagen, Denmark; vincent.vendius@sund.ku.dk (V.F.D.V.); mema@sund.ku.dk (M.M.); dbel@sund.ku.dk (D.B.); 2 ADM Denmark A/S, 3390 Hundested, Denmark; 3 Section for Oral Biology and Immunopathology, Department of Odontology, Faculty of Health and Medical Sciences, University of Copenhagen, 2200 Copenhagen, Denmark; laura.massarenti@sund.ku.dk (L.M.); chrd@sund.ku.dk (C.D.); 4 Department of Periodontology, Institute of Dentistry, University of Turku, 20520 Turku, Finland; ulvi.gursoy@utu.fi (U.K.G.); mervi.gursoy@utu.fi (M.G.); 5 Department of Oral Biochemistry, Academic Center for Dentistry Amsterdam, University of Amsterdam and VU University Amsterdam, 1081 LA Amsterdam, The Netherlands; a.p.van.splunter@acta.nl (A.V.S.); f.bikker@acta.nl (F.J.B.)

**Keywords:** dental plaque, microbiota, probiotics, randomized controlled trial, sugar stress, 16S ribosomal RNA

## Abstract

The aim was to test if probiotics counteract oral dysbiosis during 14 days of sugar stress and subsequently help restore oral homeostasis. Eighty healthy individuals received either probiotics (*n* = 40) or placebo lozenges (*n* = 40) for 28 days and rinsed with a 10% sucrose solution 6–8 times during the initial 14 days of the trial. Saliva and supragingival samples were collected at baseline, day 14, and day 28. Saliva samples were analyzed for levels of pro-inflammatory cytokines, albumin, and salivary enzyme activity. The supragingival microbiota was characterized according to the Human Oral Microbiome Database. After 14 days of sugar stress, the relative abundance of *Porphyromonas* species was significantly higher (*p* = 0.03) and remained significantly elevated at day 28 in the probiotic group compared to the placebo group (*p* = 0.004). At day 28, the relative abundance of *Kingella* species was significantly higher in the probiotic group (*p* = 0.03). *Streptococcus gordinii* and *Neisseria elongata* were associated with the probiotic group on day 28, while *Streptococcus sobrinus* was associated with the placebo group on day 14 and day 28. On day 28, the salivary albumin level was significantly lower in the probiotic group. The present study demonstrates a potential stabilizing effect on the supragingival microbiota mediated by consumption of probiotics during short-term sugar stress.

## 1. Introduction

The oral microbiota is comprised of more than 700 bacterial species, making the oral microbiota the second most complex found in the human body [1].

In oral health, the oral microbiota thrives with the human host, whereas dysbiotic compositional changes are associated with development of oral diseases [2].

Probiotics are live microorganisms, which, when administered in adequate amounts, confer a health benefit on the host [3]. The specific mechanisms of probiotics remain to be clarified in detail, but probiotics are believed to have a local effect on the oral microbiota by improving the microbiological composition, together with an indirect beneficial augmentation of the immune system [4]. In recent decades, a growing interest in alternative supportive and preventive approaches to maintain and achieve oral health has blossomed, and probiotics are, among others, suggested as a potential adjunct in the prevention of dental caries [4,5,6,7].

Free sugars constitute the most important dietary risk factor for the development of dental caries [8,9], with a dose-response relationship [10]. Cross-sectional data show that the oral microbiota associates with an intake of free sugars, as the microbiota of individuals with a high intake of free sugar is less diverse and harbors a higher abundance of aciduric and acidogenic bacterial species as compared to individuals with a low intake of free sugar [11,12]. Thus, intake of free sugar is an external perturbation to the oral ecosystem, affecting the composition of the oral microbiota, thereby fueling the transition from oral health towards dental caries [12,13]. Recently, a high intake of free sugars has likewise been suggested to be associated with increased inflammatory levels in saliva [14]. Ideally, probiotics to be used in the prevention of caries should be able to protect the oral cavity against sugar-mediated alterations, such as loss of diversity and potential fluctuations in inflammatory mediators in saliva.

Recently, we showed that intake of probiotic lozenges containing an equal mix of *Lacticaseibacillus rhamnosus* PB01 DSM14870 and *Lactilactobacillus curvatus* EB10 DSM32307, in combination with rinsing with a 5% xylitol solution twice a day for 14 days, induced significant changes to the salivary microbiota, which were antagonistic to the effect of rinsing with a 10% sucrose solution [15]. Thus, our data highlight a potential protective effect on the salivary microbiota when probiotics are consumed in combination with xylitol. However, it remains to be tested whether the abovementioned probiotic supplement has a protective effect on the supragingival microbiota during transient sugar stress and if this translates into clinical changes. Indeed, considering that high sugar consumption relates to chronic low-grade inflammation [16] and immune response dysregulation [17], the effects of probiotic supplement use during sugar stress on salivary cytokine levels and protease activities need to be characterized.

We therefore hypothesized that in a randomized, double-blinded clinical trial, a daily supplement of probiotics would counteract the detrimental effects of 14 days of sugar stress on the supragingival microbiota and help restore oral homeostasis over the subsequent 14 days, when sugar stress is terminated in systematically and orally healthy individuals. The aim of the present study was to determine if consumption of probiotic lozenges containing an equal mix of *L. rhamnosus* PB01 DSM14870 and *L. curvatus* EB10 DSM32307 and xylitol could counteract oral dysbiosis in the supragingival microbiota in systemically and orally healthy individuals incited by 14 days of sugar stress, and if the same probiotic supplement could help restore oral homeostasis during the subsequent 14 days when sugar stress was terminated. Second, the aim was to quantify the impact of daily supplementation with probiotics, during and after sugar stress, on levels of selected cytokines and proteases, as well as the plaque index and bleeding-on-probing percentage (BOP%).

## 2. Materials and Methods

### 2.1. Study Design

**Figure 1:**
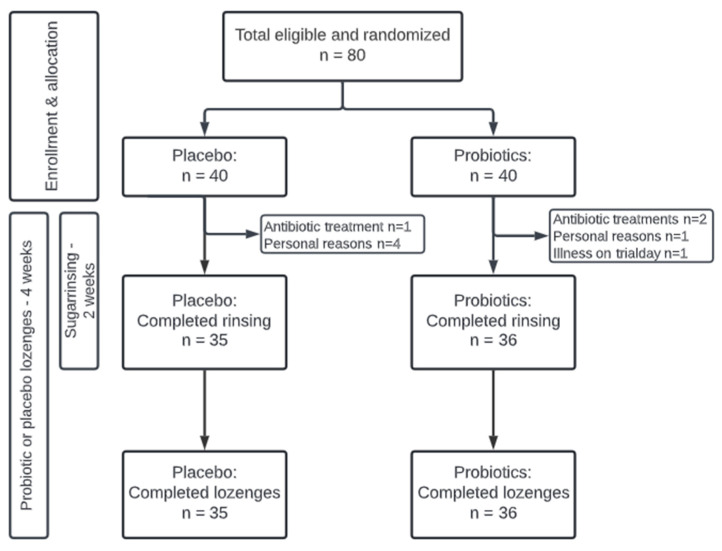
Flowchart of the study.

The present study was performed at the Department of Odontology, University of Copenhagen, from November to December 2021 as a quadruple-blinded (participant, care provider, investigator, outcome assessor), randomized, placebo-controlled, clinical trial with a total duration of 28 days. At baseline, computerized randomization (www.randomizer.org accessed on 29 October 2021) was performed by DB to allocate participants to receive either probiotic or placebo lozenges twice daily throughout the trial period. During randomization, the numbers 1–80 were allocated to either the probiotic or placebo group, whereafter DB fitted the numbers to identical-looking probiotic or placebo pots. Furthermore, all participants received a 10% sucrose solution, which they were instructed to rinse with 6–8 times a day for the initial 14 days of the trial period. Clinical examination and sample collection were performed at baseline, day 14, and day 28 and all subjects signed informed consent before participation. Normal oral hygiene procedure was followed throughout the trial. Microbial data from the placebo group have been published previously [16]. The study was approved by the regional ethical committee (H-21003295) and performed following the Helsinki Declaration. Finally, the study was registered at ClinicalTrials.gov (UCPH_01_005) and reported to the local data authorization of the Faculty of Health and Medical Sciences, University of Copenhagen (514-0434/19-3000). The timeline of the study is detailed in Figure 1.

### 2.2. Study Population

Eighty orally and systemically healthy individuals aged 19–31 years were recruited at the Department of Odontology, University of Copenhagen, by CLO. The sample size was based on a power calculation using data from our previously published paper [15], which revealed that *n* = 20 was sufficient to detect a 70% increase in Streptococcal species in saliva after 14 days of sugar stress. We estimated the impact of sugar stress to be at least 50% less on the supragingival microbiota, resulting in a sample size estimate of $n=20+\left(20\cdot 0.5\right)=30$ in each group. To ensure that sufficient power could be maintained despite dropouts of as much as 30%, we enrolled *n* = 80 (*n* = 40 per group). The inclusion criteria were systemic and oral health and age between 18–35 years, while the exclusion criteria were current tobacco use, pregnancy, and antibiotics intake three months before participation and during the intervention period.

### 2.3. Clinical Examination

Clinical examination details have previously been described [18,19]. In brief, levels of plaque and BOP% were registered at baseline, day 14, and day 28 (±2 days). Levels of plaque were registered using SUNSTAR G·U·M^®^MD RED-COTE^®^MD disclosing tablets and graduated from 0–5 by use of the Modified Quigley and Hein index [20]. All examinations were performed by the same examiner (CLO).

### 2.4. Collection of Samples

As previously described, 2 mL of paraffin-stimulated saliva was collected [15,21]. Subsequently, a supragingival plaque sample from the buccal surface of the 1st quadrant was collected as earlier performed but with slight modifications [19,22]. All samples were collected from Wednesday to Friday between 8 a.m. and 5 p.m. and great effort was made to ensure that samples were collected from the same participant at the same time of the day on the three trial days. Participants were instructed to refrain from eating and drinking two hours before sampling and to refrain from oral hygiene procedures on trial days to secure enough supragingival biofilm for sampling. Supragingival plaque samples were pooled and vortexed in 1 mL saline. All samples were immediately cooled to −18 °C and then to −80 °C within 8 h, and stored until further analyses.

### 2.5. Probiotics and Placebo

The probiotic lozenges contained an equal mix of *L. rhamnosus* PB01 DSM14870 and *L. curvatus* EB10 DSM32307 with a concentration of 1 × 10^9^ CFU/tablet and 491 mg xylitol. The probiotic strains were the same as those used in previous studies [15,19,23]. The probiotic and placebo lozenges were almost identical in size, taste, and composition, except for the probiotic strains and xylitol, which unavoidably made the probiotic lozenges sweeter. The lozenges were packed in identical pots, and participants as well as the examiner were blinded throughout the trial period. Subjects were guided through written, verbal, and video instructions to soak and distribute one lozenge twice a day, right after tooth brushing, to ensure that the probiotic strains could interfere with the initial biofilm formation. Moreover, participants were instructed to refrain from any food or drink consumption during the subsequent 30 min to avoid oral clearance and ensure longer exposure of the probiotic strains to the oral cavity. Both probiotic and placebo lozenges were produced at ADM Denmark A/S (Hundested, Denmark).

### 2.6. Sucrose Solution

An amount of 240 L of 10% sucrose solution was prepared at the University of Copenhagen, as previously described [15]. Participants were instructed to rinse with the sucrose solution 6–8 times a day through written, verbal, and video guides, which have been described previously [15].

### 2.7. DNA Extraction, Library Preparation, and DNA Sequencing

The protocol used for DNA extraction, library preparation, and 16S-sequencing has been described in detail elsewhere [15,19]. In brief, by use of the MiSeq (Illumina, San Diego, CA, USA), we targeted the V1–V3 region of the 16S gene. Failed samples were those yielding significantly less quality filtered DNA reads (filtReads) than 10,000; here, filtReads < 8000. In this project, no samples failed, and all were therefore included in analyses. Bioinformatic processing of sequence data was performed as previously described [15,18,19] by matching against the 16S rRNA Human Oral Microbiome RefSeq database (HOMD) v. 15.2 [1].

### 2.8. Cytokine Analysis

Cytokine analysis was performed as previously described [24,25]. In brief, saliva samples were thawed and centrifuged at 9300× *g* for 5 min at room temperature. The levels of interleukin (IL)-1β, IL-8, monocyte chemoattractant protein-1 (MCP-1), and macrophage migration inhibitory factor (MIF) were determined from the salivary supernatants using a bead-based immunoassay (Luminex xMAP, Luminex Corporation, Austin, TX, USA) with commercial kits (Bio-Plex Pro Human Cytokine Screening Panel, Bio-Rad Laboratories, Bio-Rad, Santa Rosa, CA, USA) according to the manufacturer’s instructions. The assays had the following limit of detection (LOD) ranges: 0.24 pg/mL for IL-1β, 0.36 pg/mL for IL-8, 2.45 pg/mL for MIF, and 0.44 pg/mL for MCP-1. For this project, measurements for 24 samples were below the LOD for MIF and therefore replaced with LOD/2. All laboratory analyses were performed blindly.

### 2.9. Protein and Enzyme Analysis

Saliva samples were centrifuged at 4 °C for 10 min at 10,000× *g* and then three aliquots were made from saliva supernatant for BCA and Amylase, total protease activity and chitinase, and for albumin to avoid freeze–thaw cycles. The samples were stored at −20 °C until further analysis.

Per the manufacturer’s instructions, the total protein concentration was analyzed as described [26,27] by PierceTM BCA Protein Assay Kit (ThermoFisher, West Palm Beach, FL, USA, Cat#23227). The saliva samples were diluted 1:4 in phosphate-buffered saline (PBS) before testing, and on each plate a bovine serum albumin (BSA) concentration series was included as a positive control and used to calculate the protein concentration in samples using BSA standard (BSA: 25–1500 ug/mL).

Amylase activity was measured in saliva by diluting the samples 1:100 in MILLI-Q and mixing them with alpha-amylase substrate consisting of 2-Chloro-4-nitrophenyl-α-D-maltotrioside (Apollo Scientific, Denton, UK, BITJ00020) as described earlier [28].

Total protease activity (TPA) was measured using PEK-54 substrate ([FITC]-NIeKKKKVLPIQLNAATDK-[KDbc]) as described elsewhere [29], with a working solution of 32 µM diluted in TBS. 50 µL of saliva, and 50 µL of PEK-54 substrate was added to a black 96-well plate (non-binding), resulting in a final PEK-54 substrate concentration of 16 uM. Reaction mixes were placed in a fluorescence microplate reader with a 485 nm excitation filter and 530 nm emission filter (gain 800) and fluorescence was measured for approximately 1 h at 37 °C using 5 min scanning intervals. Proteolytic activity was expressed as the increase in fluorescence per min (F/min).

To measure the chitinase activity, 50 µL 4-methylumbelliferyl b-D-N,N′,N″-triacetylchitotriose substrate with a final concentration of 12.7 µM was mixed with 50 µL saliva in a black 96-well plate (non-binding). In every plate, a chitinase control enzyme with a concentration of 0.001 mg/mL was used as a positive control, while a substrate without samples and assay buffer was used as a negative control. Reaction mixes were placed in the fluorescence microplate reader with a 360 nm excitation filter and 450 nm emission filter and fluorescence was measured for approximately 1 h at 37 °C using 5 min intervals.

Albumin was measured as previously described [26,27] with some modifications. In brief, microplates were coated with rabbit anti-human albumin, cat# A0001 DAKO (Glostrup, Denmark). Time point 1 dilution series of saliva samples were tested (1:500 to 1:6400) to obtain the best sample dilution factor. Later, dilution of 1:2000 was used for all samples. An albumin concentration series was included as a positive control on each plate and used to calculate the albumin concentration in samples (concentration: 0.1 µL/mL–0.00156 µL/mL). Anti-human albumin (horseradish peroxidase (HRP)), (Biorbyt via bioconnect, Cat# ORB243267) was used as conjugate and albumin was detected with OPD substrate tablets (o-phenylenediamine dihydrochloride) (Thermo Fisher #34006, West Palm Beach, FL, USA) according to protocol.

### 2.10. Bioinformatic Processing and Statistics

Clinical data and plaque and bleeding index were compared between groups and sampling times by multiple linear regression and adjusted for baseline levels (ANCOVA) in R (version 4.2.3) using R studio (IDE version 2023.04.1 + 446).

The supragingival microbiota was characterized and compared by relative abundance and visualized by principal component analysis (PCA), while statistically significant differences in microbial community compositions were tested by linear discriminant analysis effect size (LEfSe). Data on relative abundance were corrected for multiple dependent associations using Benjamini—Hochberg correction [30]. Bioinformatic processing and statistics on the supragingival microbiota were performed in R v. 4.1.0 through the R studio (IDE version 1.4.1717 by use of the ampvis package v.2.7.8 [31] as described in our previous studies [15,19]. Alpha diversity was adjusted for baseline values and compared using linear regression (ANOVA) on day 14 and day 28 using R (version 4.3.0) and R studio (IDE version 2023.06.0 + 421).

Differences in cytokines, albumin, and enzyme activity between groups were analyzed by ANCOVA on log10-transformed data adjusted for baseline levels, using R (version 4.2.2) and R studio (IDE version 2023.06.2 Build 561). A *p*-value ≤ 0.05 was considered significant for all analyses.

## 3. Results

### 3.1. Background and Clinical Data

The study population consisted of 80 systemically and orally healthy individuals. Seventy-one individuals completed the trial distributed equally between the probiotic (*n* = 36) and placebo groups (*n* = 35). Nine participants dropped out during the trial period due to personal reasons (*n* = 5), antibiotic prescription (*n* = 3), and illness (*n* = 1). All dropouts were within the first 14 days of the intervention and were consequently excluded from further analyses. There was an overrepresentation of females in the trial with 29/36 in the probiotic group and 26/35 in the placebo group. Likewise, most of the participants in both the probiotic group (*n* = 21) and the placebo group (*n* = 25) were dental students. The mean age was 24 years in the probiotic group with a range of 20–30 years, and 23.4 years in the placebo group with a range of 19–30 years. Clinical data of plaque score and BOP% are presented in Table 1. No significant differences were observed at any time points between groups.

### 3.2. Sequencing Metadata

Our 213/213 sample analyses (100%) were successful for DNA extraction and sequencing library preparation. The sample analyses yielded between 42,602 and 202,974 DNA reads after quality check and bioinformatic processing, therefore 42,602 reads per sample were included in downstream analysis. From the 213 samples, 17 million reads were retrieved and 3238 unique operational taxonomic units (OTUs) were identified. A total of 95 different bacterial genera were identified corresponding to 91.48% coverage of the generated sequences, while 339 different bacterial species were identified corresponding to 59.94% coverage of the generated sequences. Determined by the Shannon and Simpson indices, the mean alpha diversity across all 213 samples was 4.12 (2.56 to 5.4) and 3.28 (1.10 to 4.72), respectively.

### 3.3. Probiotics Partly Protect against Sugar-Mediated Loss of α-Diversity

**Figure 2:**
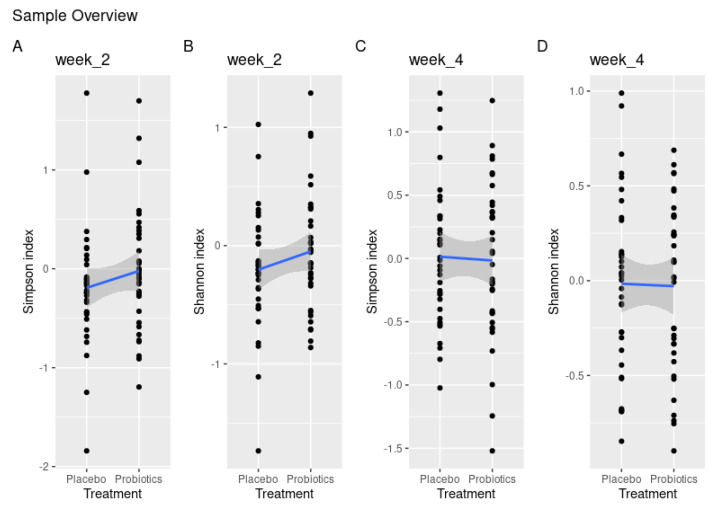
Treatment effect measured by disparities in α-diversity demonstrated by Shannon and Simpson indices between placebo and probiotics after 14 days of sugar stress (**A**,**B**) and 14 days after discontinuation of sugar stress at day 28 (**C**,**D**).

Intragroup comparison of α-diversity showed a significant loss of diversity from baseline to day 14 in the placebo group with the Shannon index (*p* = 0.02), which was borderline significant with the Simpson index (*p* = 0.06). This loss was not visible in the probiotic group (Shannon index, *p* = 0.6 and Simpson index, *p* = 0.1). Yet, when comparing α-diversity between groups, this difference failed to reach significance using both the Simpson and Shannon indices (*p* = 0.23, *p* = 0.2) (Figure 2A,B). On day 28, there were no noteworthy differences in α-diversity using either the Simpson or Shannon index (*p* = 0.83, *p* = 0.91) (Figure 2C,D).

### 3.4. Compositional Changes of the Supragingival Microbiota

**Figure 3:**
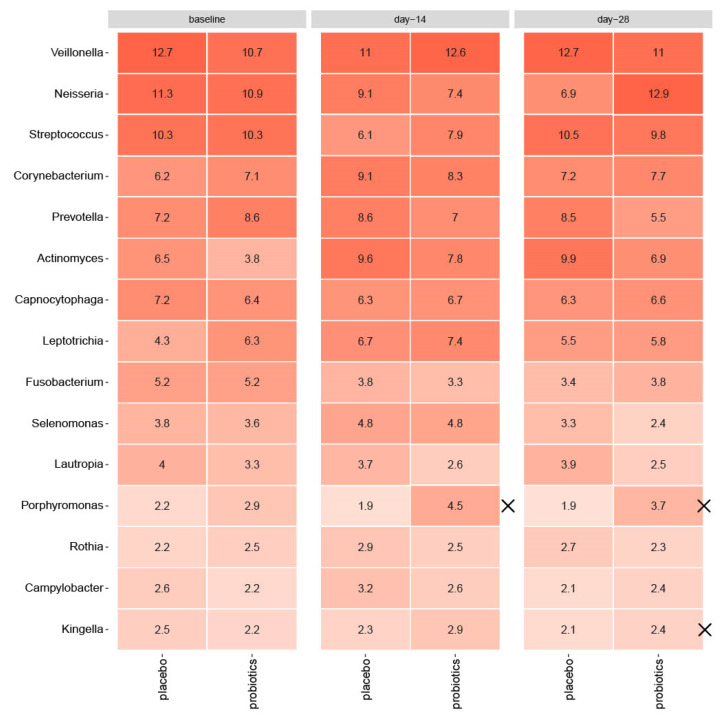
Impact of sugar stress on predominant microbiota for the placebo and probiotic groups, respectively, and mean values of relative abundance of 15 predominant genera. X marks significant differences between groups. Intensity of coloration marks increasing abundance.

As shown in Figure 3, sugar stress caused a significant increase in *Actinomyces* species (6.5% to 9.6% for placebo, *p* = 0.006, and 3.8% to 7.8% for probiotics, *p* = 0.002) from baseline till day 14. In the placebo group, sugar stress furthermore caused a significant increase in *Corynebacterium* species (6.2% to 9.1%, *p* = 0.03), together with a decrease in *Streptococcal* species (10.3% to 6.1%, *p* = 0.001), which was not evident in the probiotic group (*p* = 0.2 for *Corynebacterium* species, *p* = 0.1 for *Streptococcal* species). In the probiotic group, *Fusobacterium* species significantly decreased during sugar stress (5.2% to 3.3%, *p* = 0.04), which was only borderline significant in the placebo group (*p* = 0.06). Nevertheless, none of the observed differences were significant between groups at day 14 (*Actinomyces* species *p* = 0.3, *Corynebacterium* species *p* = 0.9, *Streptococcal* species *p* = 0.2, *Fusobacterium* species *p* = 0.7). *Actinomyces* species remained elevated at day 28 (6.5% to 9.9% for placebo, *p* = 0.003 and 3.8% to 6.9% for probiotics, *p* = 0.0002), but the difference between groups was not significant (*p* = 0.2). Between groups, relative abundance of *Porphyromonas* species was significantly higher in the probiotic group at day 14 (*p* = 0.03) and remained significantly elevated at day 28 in the probiotic group compared to the placebo group (*p* = 0.004). At day 28, relative abundance of *Kingella* species was likewise significantly higher in the probiotic group (*p* = 0.03) compared to the placebo group. At day 28, relative abundance of *Neisseria* species was borderline significantly elevated in the probiotic group compared to the placebo group (*p* = 0.08).

**Figure 4:**
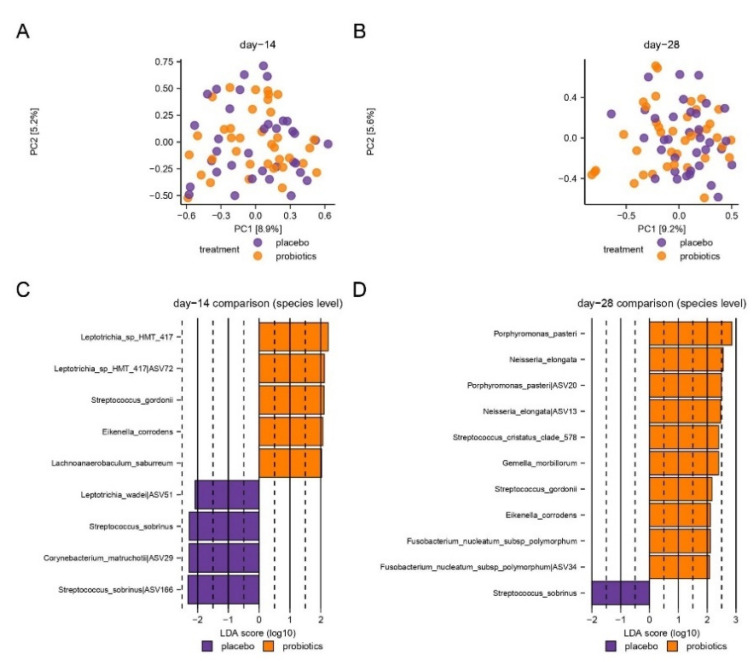
Compositional changes induced by sugar stress. Principal component analysis (PCA) is expressed by the two most decisive components (PC1 and PC2), which covered approximately 15% of the variation of the dataset. Placebo vs. probiotics day 14 (**A**). Placebo vs. probiotics day 28 (**B**). LEfSe analysis expressed by significant species at day 14 (**C**) and day 28 (**D**).

PCA based on the two most decisive components (PC1 and PC2), which covered approximately 15% of the dataset, showed an almost completely random distribution of samples collected after 14 days of sugar stress in the probiotic- and placebo groups (Figure 4A). Likewise, no evident separation of day 28 samples from the probiotics and placebo groups was observed, although a tendency of clustering was visible (Figure 4B).

LEfSe identified 13 species (Figure 4C,D), which were significantly different in the probiotic group versus the placebo group. Specifically, on day 28, *Streptococcus gordinii* and *Neisseria elongata* were associated with the probiotic group. In contrast, *Streptococcus sobrinus* was associated with the placebo group on both day 14 and day 28.

### 3.5. Salivary Levels of Pro-Inflammatory Cytokines and Proteases

Mean salivary levels of IL-1β, IL-8, MIF, and MCP-1 are presented in Table 2. No significant differences were observed at any time points between groups. Likewise, as presented in Table 3, supplementary consumption of probiotics during the trial had no significant impact on the salivary levels of chitinase, amylase, and TPA. On the other hand, the salivary level of albumin was significantly lower in the probiotic group at day 28 compared to the placebo group, after adjusting for baseline values.

## 4. Discussion

In the present study, data suggested that probiotics partly counteract sugar-mediated loss of diversity in the supragingival microbiota, which was observed in the placebo group. Consequently, from a microbiological point of view, data confirmed the hypothesis that the probiotic supplement augmented resilience of the supragingival microbiota in the resolution period after sugar stress. Importantly, data from a recent systematic review reported lower bacterial diversity in both supragingival plaque and saliva in individuals with a high dietary sugar intake, compared to individuals with a low dietary sugar intake [12]. In line with this finding, studies have shown that the supragingival microbiota in caries lesions are less diverse than the microbiota on healthy teeth surfaces [32,33,34], and the salivary microbiota from healthy individuals shows higher diversity than the salivary microbiota from individuals with dental caries [35]. Consequently, it has been thoroughly documented that loss of diversity is a central microbiological event in dental caries. Hence, as the tested probiotic supplement was partly able to prevent sugar-mediated loss of diversity, these supplements may offer a potential clinically relevant adjunct to conventional caries prevention in individuals with frequent carbohydrate consumption.

Interestingly, intake of probiotics during sugar stress partially protected against fluctuations in the supragingival microbiota and supported restoring baseline conditions of the microbiota in the resolution period after transient sugar stress (Figure 4). Specifically, the decrease of *Streptococcal* species from 10.3% to 7.9% (*p* = 0.1) observed after 14 days of sugar stress in the probiotic group was only 50% of the decrease observed in the placebo group from 10.3% to 6.1% (*p* = 0.001) (Figure 3). In addition, from baseline to 14 days after discontinuation of sugar stress, the abundance of *Neisseria* decreased from 11.3% to 6.3% in the placebo group (*p* = 0.07), while the abundance of *Neisseria* returned to baseline conditions (from 10.9% at baseline to 12.9% at day 28) in the probiotic group at day 28 (*p* = 0.8) (Figure 3). These differences were only borderline significant when adjusted for Benjamini—Hochberg correction for multiple testing and not significant when compared between groups (*Streptococcal* species *p* = 0.2, *Neisseria* species *p* = 0.08) although there was a trend toward efficacy for *Neisseria* at day 28. However, the trends are clinically interesting, as the majority of *Streptococcal* and *Neisseria* species are associated with oral health [36,37]. Thus, our data point towards the conclusion that frequent intake of the tested probiotics could potentially reduce the ecological niche for *Streptococcal* species and augment recolonization of *Neisseria* species. Yet, future studies are needed to clarify whether this impact is protective against the development of dental caries.

*S. sobrinus* was significantly more abundant in the placebo group versus the probiotic group after 14 days of sugar stress (*p* = 0.004) and after 14 days of discontinuation of sugar stress (*p* = 0.008), respectively (Figure 4C,D). *S. sobrinus* is associated with dental caries and is closely related to the most prominent cariogenic species, *Streptococcus mutans* [32,38,39,40,41]. In contrast, a significantly higher abundance of *S. gordonii* was recorded in the probiotic group after 14 days of sugar rinsing (*p* = 0.008) and was again recorded in the 14 days without sugar stress (*p* = 0.03). In addition, a significantly higher abundance of *N. elongata*, and *Porphyromonas pasterii* was noted 14 days after discontinuation of sugar stress (Figure 4C,D). *S. gordonii* is an initial colonizer of the oral cavity and is associated with oral health [42]. Furthermore, *S. gordonii* catabolizes arginine to ammonia, potentially raising the pH value [43,44], and inhibits biofilm formation and bacteriocin production by *S. mutans* [45,46]. In a recent study, a significantly higher abundance of *N. elongata* was observed in supragingival plaque samples from caries-free individuals, as compared to individuals with active caries [47], while a high abundance of *P. pasterii* in saliva recently has been associated with lower susceptibility to dental caries [48]. In general, the species associated with the probiotic group are not associated with dental caries, but rather with oral health, suggesting that the intake of probiotics during sugar stress limits the propagation of cariogenic species, such as *S. sobrinus*, and promotes the recolonization of health-associated bacterial species in the resolution period after sugar stress. Thus, probiotic supplements may strengthen the resilience of the supragingival microbiota during and after sugar stress.

We observed minimal impact of sugar stress on salivary cytokine levels and protease activity, with no clear differences between groups at any time point apart from a significantly lower level of albumin in the probiotic group at day 28 (Table 2 and Table 3). This finding is interesting as a decrease might reflect reduced leakage of plasma proteins into the oral cavity, which supports the supplementary use of probiotics [49]. Besides the decrease in albumin, the stability in salivary enzymes and cytokine levels was probably the consequence of the fact that BOP%was very low in both groups during the trial, as the participants performed meticulous oral care throughout the study period (Table 1). In line with this, the stability of chitinase and protease over time indicates mucosal stability, as their levels are known to be elevated in cases of periodontal diseases [50,51]. Accordingly, the impact of sugar stress was observed on the composition of the supragingival plaque but not the amount of plaque, which is the main effector in early inflammatory response. This finding indicates that changes in abundance are not enough to cause any inflammatory response during short-term sugar stress in a healthy oral cavity. Finally, it is important to note that in this trial the perturbation was increased sugar availability, not increased sugar intake, which hampers the possibility of characterizing the systemic effect of frequent sugar intake. It would therefore be interesting in future trials to test the effect of frequent sugar intake on oral inflammation.

Several limitations apply to the present study, including the short-term study design, which makes it impossible to address the effect of the probiotics on dental caries, as dental caries is a slow-progressing disease that usually takes months to manifest clinically. With an eye on the central role of carbohydrates in the pathogenesis of dental caries, we did not consider it ethically acceptable for the participants to perform sugar rinsing for a longer period than 14 days. It is important to note that oral hygiene was pristine in both groups and that participants, in general, reported an increased need for oral hygiene due to the increased feeling of plaque formation during sugar stress. The pristine oral hygiene in this trial hampers the possibility of extrapolating data from the present study to individuals with questionable oral care and the presence of treatment requiring oral disease. However, given the central role of frequent carbohydrate intake as a major risk factor of dental caries [8,9], and lessons learned from the Vibeholm study [52], we only considered it ethically acceptable to conduct the present study in an orally healthy population with excellent self-performed oral care.

The main strengths of the study were the large sample size with multiple sampling from each participant, and that we used 16S sequencing to characterize the composition of the supragingival microbiota, rather than focusing on the abundance of a limited number of preselected potential pathogens. Future longitudinal studies in populations such as children and adolescents, where some members inevitably develop dental caries over time, is the obvious next step needed to test the clinical relevance of the significant microbiological impact observed in the present study. Indeed, this is of particular importance, since data from studies that previously investigated the clinical effect of probiotics on dental caries have in general been inconclusive [4,5,6,7]. Despite the considerable sample size, post hoc analysis of immunological data indicate that the present study was underpowered with regards to inflammatory markers, as data on IL1- β and IL-8 were significantly different between groups with twice the sample size. Importantly, the sample size in the present study was made from a power calculation based on microbiological findings from our previous studies. As such, data from the present study underline that future studies testing the effect of probiotics on salivary levels of cytokines and salivary enzyme activity need additional power, as compared to trials with microbial endpoints.

## 5. Conclusions

In conclusion, the present study is the first to demonstrate the effect of probiotics on the composition of the supragingival microbiota during short-term sugar stress in healthy individuals. Specifically, the intake of probiotics stabilized bacterial diversity, induced partial resistance against fluctuations, and augmented the resilience of the supragingival microbiota. Future studies with larger sample sizes are needed to elucidate the clinical and immunological impact of sugar stress, while long-term studies are needed to reveal the potential clinical impact of these probiotics in the context of dental caries.

## Figures and Tables

**Table 1:** Background information of the study population. Clinical data measured by plaque and BOP% by the mean and standard deviation (sd) for placebo and probiotics at baseline, day 14, and day 28.

	Placebo (*n* = 35)	Probiotic (*n* = 36)
Sex (female/male)	26/9	29/7
Age (mean, range)	23.4 (19–30)	24 (20–30)
Dental students	25/35	21/36
Clinical examination		
Baseline		
Plaque mean (sd)	1.72 (0.3)	1.75 (0.3)
BOP% mean (sd)	2.82 (2.8)	3.10 (2.9)
Day 14		
Plaque mean (sd)	1.57 (0.3)	1.69 (0.3)
BOP% mean (sd)	3.37 (2.9)	4.18 (3.2)
Day 28		
Plaque mean (sd)	1.55 (0.3)	1.57 (0.3)
BOP% mean (sd)	3.47 (2.7)	3.94 (3.4)

**Table 2:** Salivary levels of IL-1β, IL-8, MIF, and MCP-1 expressed in pg/mL. Data are presented as the mean and standard deviation (sd) for the probiotic and placebo groups and *p*-values and confidence intervals (CI) are presented from comparison by groups performed on log10-transformed data via ANCOVA, with baseline values as covariate.

	IL-1β	IL-8	MCP-1	MIF
Baseline				
Placebo mean (sd)	1.06 (0.53)	2.2 (0.43)	1.41 (0.43)	1.61 (0.75)
Probiotics mean (sd)	1.05 (0.44)	2.17 (0.37)	1.51 (0.39)	1.75 (0.6)
Day 14				
Placebo mean (sd)	1.23 (0.48)	2.26 (0.41)	1.48 (0.42)	1.72 (0.77)
Probiotics mean (sd)	1.11 (0.46)	2.14 (0.42)	1.54 (0.43)	1.69 (0.65)
*p*-value (CI)	0.10 (−0.26; 0.03)	0.19 (−0.23; 0.05)	0.79 (−0.16; 0.12)	0.46 (−0.40; 0.18)
Day 28				
Placebo mean (sd)	1.31 (0.52)	2.28 (0.45)	1.50 (0.44)	1.78 (0.77)
Probiotics mean (sd)	1.17 (0.51)	2.14 (0.44)	1.49 (0.50)	1.62 (0.97)
*p*-value (CI)	0.11 (−0.29; 0.03)	0.14 (−0.27; 0.04)	0.30 (−0.25; 0.08)	0.16 (−0.61; 0.10)

**Table 3:** Salivary levels of amylase activity, chitinase activity, total protease activity (TPA), and albumin concentration measured by slope/min, dF/dT, slope/sec and U/mL, and µg/mL, respectively. Data are presented as the mean and standard deviation (sd) for the probiotic and placebo groups and *p*-values and confidence intervals (CI) are presented from comparison by groups performed on log10-transformed data via ANCOVA, with baseline values as covariate.

	Amylase Activity (slope/min)	Total Protease Activity (dF/dT)	Chitinase Activity (slope/s)	Albumin (µg/mL)
Baseline				
Placebo mean (sd)	0.04 (0.02)	3.12 (0.24)	0.35 (0.19)	1.77 (0.3)
Probiotics mean (sd)	0.04 (0.02)	3.13 (0.22)	0.3 (0.18)	1.74 (0.25)
Day 14				
Placebo mean (sd)	0.04 (0.02)	3.16 (0.27)	0.31 (0.17)	1.65 (0.26)
Probiotics mean (sd)	0.04 (0.02)	3.11 (0.23)	0.27 (0.16)	1.61 (0.6)
* p * -value (CI)	0.87	0.25	0.72	0.63
	(−0.005; 0.007)	(−0.16; 0.04)	(−0.03; 0.05)	(−0.13; 0.08)
Day 28				
Placebo mean (sd)	0.04 (0.02)	3.17 (0.26)	0.31 (0.16)	1.68 (0.32)
Probiotics mean (sd)	0.04 (0.02)	3.11 (0.20)	0.25 (0.16)	1.54 (0.25)
* p * -value (CI)	0.32	0.18	0.57	0.02
	(−0.003; 0.009)	(−0.15; 0.03)	(−0.05; 0.03)	(−0.23; −0.02) *

* Significant differences between groups.

## Data Availability

Raw sequences have been deposited in the European Nucleotide Archive (ENA, www.ebi.ac.uk, accessed on the 8 November 2023) with the accession number PRJEB58919. Raw cytokine and protease data are available upon request from the corresponding author. The full trial protocol can be accessed in Danish upon request from the corresponding author.
